# Angiogenesis in adipose tissue and obesity

**DOI:** 10.1007/s10456-022-09848-3

**Published:** 2022-07-20

**Authors:** Silvia Corvera, Javier Solivan-Rivera, Zinger Yang Loureiro

**Affiliations:** 1 Program in Molecular Medicine, UMass Chan Medical School, 373 Plantation Street, Worcester, MA 01605, USA; 2 Diabetes Center of Excellence, UMass Chan Medical School, Worcester, MA, USA; 3 Morningside Graduate School of Biomedical Sciences, UMass Chan Medical School, Worcester, MA, USA

**Keywords:** Endothelial, Adipocyte, ADSC, Diabetes, Hypoxia, Collagen, Extracellular matrix

## Abstract

While most tissues exhibit their greatest growth during development, adipose tissue is capable of additional massive expansion in adults. Adipose tissue expandability is advantageous when temporarily storing fuel for use during fasting, but becomes pathological upon continuous food intake, leading to obesity and its many comorbidities. The dense vasculature of adipose tissue provides necessary oxygen and nutrients, and supports delivery of fuel to and from adipocytes under fed or fasting conditions. Moreover, the vasculature of adipose tissue comprises a major niche for multipotent progenitor cells, which give rise to new adipocytes and are necessary for tissue repair. Given the multiple, pivotal roles of the adipose tissue vasculature, impairments in angiogenic capacity may underlie obesity-associated diseases such as diabetes and cardiometabolic disease. Exciting new studies on the single-cell and single-nuclei composition of adipose tissues in mouse and humans are providing new insights into mechanisms of adipose tissue angiogenesis. Moreover, new modes of intercellular communication involving micro vesicle and exosome transfer of proteins, nucleic acids and organelles are also being recognized to play key roles. This review focuses on new insights on the cellular and signaling mechanisms underlying adipose tissue angiogenesis, and on their impact on obesity and its pathophysiological consequences.

## Functions of adipose tissues

The defining feature of adipose tissue is the presence of adipocytes, which have the unique capacity to accumulate large amounts of lipid within specialized droplets, and give adipose depots their characteristic appearance. Despite a similar morphology, adipocytes comprise a heterogenous population of cells, and different types of adipocytes in each depot enable specialized functions [1–3]. For example, thermogenic adipocytes in adipose depots that surround central organs play a critical role in maintaining core body temperature. In another example, subcutaneous adipose tissue (SqAT), the largest depot, is best able to expand in response to increased food intake. Other depots are more restricted in their ability to expand, and have other functions, including immunological surveillance by mesenteric adipose tissue [4], and regulation of vessel tone by perivascular adipose tissue [1]. The roles of other adipose tissue depots, such as those in the bone marrow, are still being defined [5, 6].

In adults, SqAT mass is closely associated with metabolic disease risk. Paradoxically, the ability to expand superficial SqAT, particularly in the lower body, is associated with a *lessened* risk of metabolic disease. In the early 1980s Smith et al. analyzed 930 men and women and concluded that, for a given body mass index (BMI), visceral adiposity, reflected by waist circumference, was associated with increased insulin resistance and risk of developing type 2 diabetes and cardiovascular disease [7]. However, it is now recognized that visceral adiposity is a surrogate for the many sites within the body that accumulate fat when the SqAT cannot expand. In a recent review, Piche et al. [8] discuss the idea that pathogenic obesity, defined as causing prejudice to health, can no longer be evaluated solely by the body mass index. Even individuals who have normal weight are at high risk of metabolic disease if they accumulate fat in normally lean tissues such as liver, heart, and skeletal muscle. Conversely, individuals who are overweight or obese can be at much lower risk of metabolic disease if they have the ability to expand their SqAT mass, particularly in the gluteal-femoral area, and therefore protect other organs [8, 9].

The prevailing hypothesis explaining the paradoxical association between increased superficial SqAT mass and protection from metabolic disease is that this depot is optimally suited to store lipids. Under chronic caloric excess, energy is converted into fat through de-novo lipogenesis in the liver, and is transported via lipoproteins to adipose tissue for storage. In the absence of sufficient functional SqAT, fat accumulates in other adipose depots and in lean tissues, leading to cellular stress, inflammation and insulin resistance, setting the stage for the development of metabolic disease [10].

The urgency of understanding and mitigating metabolic disease is high; global deaths and disability-adjusted life years doubled from 1990 to 2017 [11], and a recent study found that genetically predicted higher BMI was associated with increased risk of type 2 diabetes mellitus, multiple circulatory disease outcomes including ischemic heart disease, asthma, chronic obstructive pulmonary disease, five digestive system diseases including non-alcoholic liver disease, three musculoskeletal system diseases, and multiple sclerosis as well as cancers of the digestive system (six cancer sites), uterus, kidney, and bladder [12]. Understanding the factors that control the development of SqAT and its capacity to protect lean organs from lipotoxicity is critically important in our efforts to mitigate metabolic disease pathogenesis.

## Mechanisms of adipose tissue expansion

**Fig. 1:**
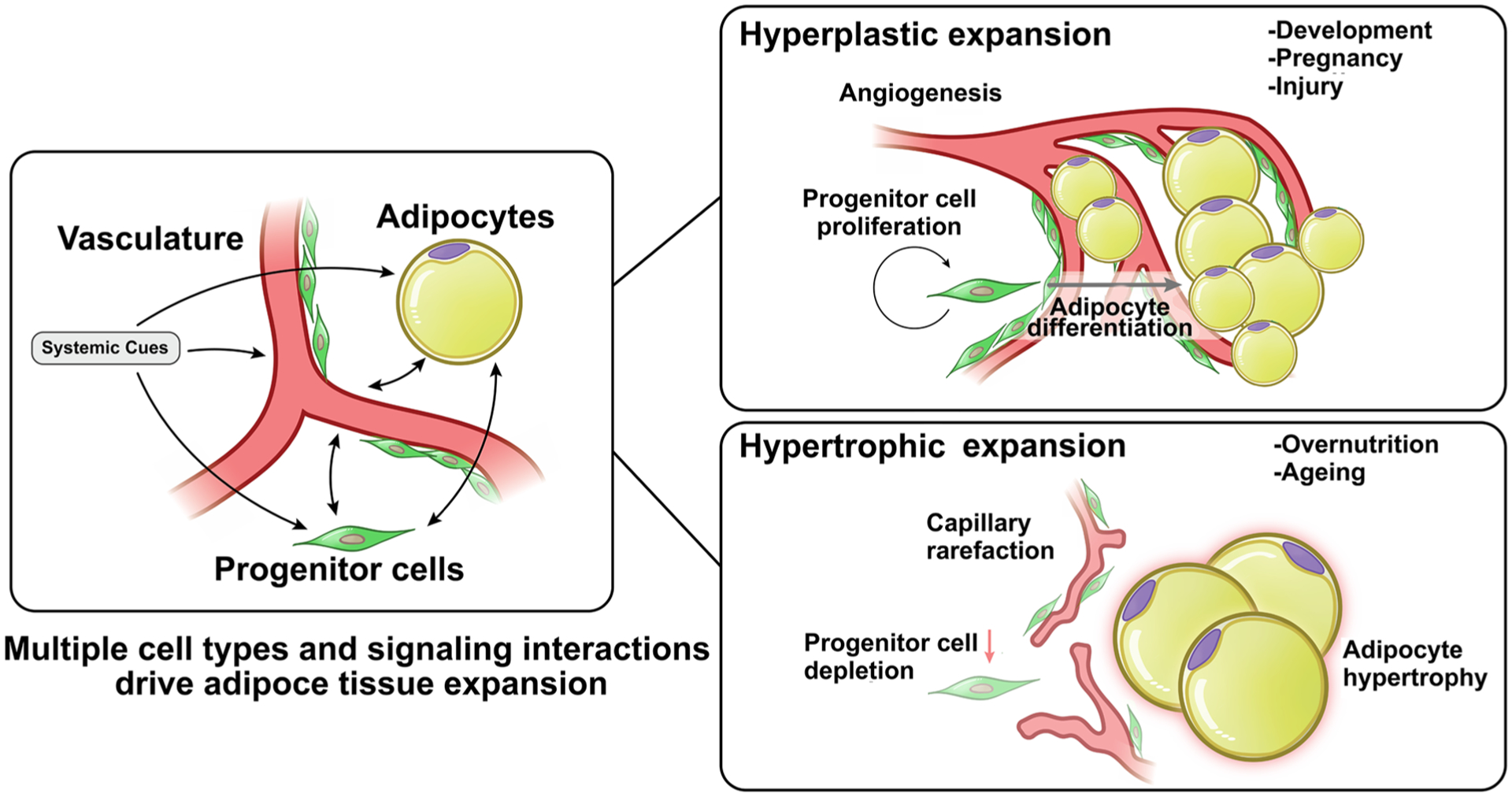
Modes of adipose tissue expansion. Adipose tissue expansion involves numerous cell types, including endothelial cells of the vasculature, adipocytes and multipotent progenitor cells. Under some conditions, angiogenesis, progenitor cell proliferation and adipocyte differentiation lead to hyperplastic expansion, forming tissue containing multiple small adipocytes. Under other conditions, angiogenesis fails, leading to capillary rarefaction, impaired multipotent progenitor cells proliferation and adipocyte hypertrophy. Hypertrophic expansion is strongly associated with metabolic disease risk

Adipose tissue can expand through two different mechanisms: hyperplasia and hypertrophy. Hyperplasia entails the de-novo differentiation of adipocytes from progenitor cells, resulting in an enlarged adipose tissue mass comprised of numerous, small adipocytes (Fig. 1). A second mechanism of adipose tissue expansion is through hypertrophy, where the size of each existing adipocyte increases to accommodate increasing amounts of fuel, resulting in an enlarged tissue mass composed of fewer, large adipocytes. These two very different expansion modes predominate under different circumstances; hyperplastic adipose tissue expansion occurs mostly during development, and is likely to be defined by genetic mechanisms that influence the number of progenitor cells and their ability to differentiate into appropriate adipocyte subtypes [13]. Hypertrophic expansion occurs mostly post-developmentally, and is dependent on the ability of existing adipocytes to capture and retain circulating lipids [14]. As discussed below, however, both hyperplasia and hypertrophy can contribute to adipose tissue expansion in adults, and the predominance of each mechanism can vary between individuals and between depots. Importantly, the degree to which hypertrophy predominates is associated with higher metabolic disease risk [15].

Hyperplastic and hypertrophic growth must be accompanied by concomitant increases in supporting structures, including vasculature and innervation, but the mechanisms underlying angiogenic expansion during hyperplastic or hypertrophic growth are fundamentally different. Critically, as detailed below, angiogenesis *precedes* hyperplastic expansion, ensuring an adequate blood supply to the developing tissue, but *follows* hypertrophic adipocyte growth and is often insufficient, leading to hypoxia and tissue dysfunction [16, 17]. The mechanisms of angiogenesis operating under either hyperplastic or hypertrophic adipose tissue growth must be considered when evaluating the effects of pro- or anti-angiogenic perturbations on adipose tissue mass and its consequences to systemic metabolism. As discussed further below, suppression of angiogenesis during development, or during hyperplastic or hypertrophic growth in adults, could have very different effects on adipose tissue composition and function. Thus, understanding angiogenic mechanisms accompanying hyperplastic or hypertrophic expansion is critical for conceptualizing therapeutic approaches to metabolic disease centered on adipose tissue angiogenesis.

## Angiogenesis during hyperplastic adipose tissue expansion

Adipose tissue development during embryogenesis involves the emergence of lipid-laden adipocytes differentiating from progenitor cells from distinct embryonic layers [18]. In multiple species including pig, rodents and humans, distinct stages of adipose tissue development have been delineated: First proliferation of primitive blood vessels is seen within loose connective tissue, second, mesenchymal cells within the vascular matrix accumulate fine fat vacuoles in their cytoplasm, and finally, clear adipocytes and fat lobules are formed [19–21]. More recent morphological analysis of epicardial adipose tissue during human development [22] reveals similar stages, with the appearance of mesenchyme at 33–35 gestational days, followed by angiogenesis at 42–45 gestational days, followed by appearance of multilocular adipocytes in primitive fat lobules, and finally by appearance of unilocular adipocytes in definitive fat lobules. Thus, in all morphological studies of adipose tissue development, including multiple species and depots, angiogenesis precedes the appearance of lipid-laden adipocytes.

Despite its predominance during development, hyperplastic adipose tissue expansion can also occur in adult animals. In some depots, for example the mouse epididymal fat pad, adipocyte formation continues after birth and is also preceded by rapid expansion of a dense vascular network within the tip of the fat pad [23, 24]. The cellularity of the developing tissue is determined by the rate of proliferation of adipocyte progenitors, which is responsive to dietary lipid composition [13]. In another example, the expansion of the interscapular brown fat depot in rodents in response to cold acclimation involves the generation of new brown adipocytes, and is critically dependent on sympathetic nerve activity and angiogenesis [25, 26]. In a different context, sympathetic innervation has been shown to activate the angiogenic switch that fuels exponential tumor growth [27]. Analogously, the adrenergic response to cold may induce an angiogenic switch that allows proliferation and differentiation of brown adipocyte progenitors, but this has not been confirmed in either mice or humans.

The interdependency between blood vessel expansion and hyperplastic adipose tissue expansion can be explained by lineage tracing studies revealing that adipocyte progenitors are found to be tightly associated with the vasculature [28–33]. Direct observation of embryonic mouse adipose tissue development also revealed proliferating preadipocytes residing as clusters distributed along the growing adipose vasculature [34]. A functional relationship between the microvasculature development and adipocyte progenitor proliferation is supported by the finding that culture conditions that promote angiogenesis also promote the proliferation of mesenchymal progenitors, which are tightly associated with emergent capillary sprouts [35, 36].

In human adults, the capacity for adipocyte hyperplasia varies markedly between depots and between subjects. For example, in response to experimental overfeeding in healthy, normal-weight adults abdominal SqAT adipocyte size increased and correlated with relative upper-body fat gain, but lower-body fat responded by hyperplasia [15]. Because measurements of adipocyte size in human tissue samples can vary dependent on histological technique, hyperplasia is defined as the proportion of adipocytes falling in the lower distribution of adipocyte size, relative to total body adiposity [37–42]. A positive correlation between adipocyte hyperplasia and metabolic health has been observed in many studies [43–45].

The mechanisms underlying individual variation in hyperplastic adipose tissue expansion are not known, but angiogenic factors may play critical roles. Genes involved in angiogenic patterning affect adipose tissue function [46]; for example, forkhead box C2 (FOXC2) in adipose tissue affects angiogenesis and vascular patterning [47], and is associated with adipose tissue function and obesity [48]. The apelin/apelin receptor signaling pathway, discovered as a vasculogenic guiding factor in zebra fish [49], is an important regulator of insulin sensitivity, glucose utilization and diabetes risk [50–52], and indirect evidence suggests apelin regulates adipose tissue vasculature and adipocyte size [53].

In addition to developmental patterning, adipose tissue cellularity is responsive to systemic signals that regulate growth and metabolic needs of the body. Among the central mechanisms that regulate body size and metabolism is the insulin-like growth factor-1 (IGF-1) signaling pathway [54]. IGF-1 is expressed around capillaries, in small fat cells, and in fibroblasts in fetal adipose tissue [55], and strongly stimulates endothelial cell proliferation and angiogenesis [56–61]. In vivo, mice in which insulin and IGF-1 signaling were abrogated in a tissue-selective manner demonstrated a critical need for IGF-1 signaling for adipose tissue development [62]. Insight into the role of IGF-1 in human adipose tissue comes from patients with deficiency of growth hormone (GH), which induces IGF-1 [63]. Adipose tissue of adult individuals with GH deficiency contain extremely large adipocytes, accompanied by decreased tissue concentration of vascular endothelial growth factor-A (VEGFA), stromal cell-derived factor (SDF1), angiopoietin 2 and BDNF, consistent with impaired angiogenesis [40]. Similarly, children with GH deficiency have an increased mean adipocyte volume but a reduced number of fat cells which normalized upon GH treatment [64]. In vitro, inhibition of IGF-1 receptor signaling completely abrogates proliferation of mesenchymal progenitors and endothelial cells from human adipose tissue [56].

Special conditions of hyperplastic adipose tissue growth in adults are pregnancy [65] and injury, for example following myocardial infarction. Adipose tissue hyperplasia in pregnancy is associated with placental mechanisms regulating adipose tissue IGF-1 bioavailability, which are associated with protection from gestational diabetes [56, 66]. Generation of epicardial adipose tissue following injury has been found to be critically dependent on IGF-1 receptor signaling [67]. In aggregate, in vitro and in vivo evidence in both mice and humans points of a critical role of IGF-1 signaling in mediating adipose tissue angiogenesis and hyperplastic adipose tissue expansion.

## Angiogenesis and hypertrophic adipose tissue expansion

In the absence of hyperplastic expansion, excess caloric intake leads to adipocyte hypertrophy (Fig. 1). In mice, high calorie, high fat diets or hyperphagia lead to adipocyte hypertrophy in the epidydimal adipose depot [68–70], which is accompanied by disruption in capillary architecture and blood flow, leading to hypoxia [71]. Similar thinning and disruption of blood vessel architecture is seen in response to high calorie, high fat diet in mouse brown fat [72]. Adipocyte hypertrophy in response to overfeeding is also seen in humans, observed preferentially in abdominal adipocytes [37, 73–75]. Most studies find that increased adipocyte size is accompanied by capillary rarefaction in both SqAT and visceral adipose tissue (VAT), and is enhanced in subjects with Type 2 diabetes mellitus (T2DM) [74, 76]. In general, the degree of hypertrophy of abdominal adipocytes correlated inversely with metrics of insulin sensitivity.

The studies above concur in finding a strong correlation between adipocyte hypertrophy and impaired capillary architecture, but the cause-effect associations are not understood. One possibility is that adipocyte hypertrophy causes capillary insufficiency, by for example by failing to elicit adequate angiogenic signaling or by creating a niche that is non-permissive for angiogenesis, resulting in capillary rarefaction relative to adipose mass [77–79]. Alternatively, high caloric intake may directly disrupt angiogenic pathways and capillary function, and these alterations may cause adipocyte hypertrophy, for example by limiting removal of fatty acids from the extracellular space. A third possibility is that overfeeding affects both adipocyte and the capillary functions, leading to the observed phenotype of enlarged adipocytes and disrupted capillary networks. Mechanistically, VEGF-A165b has been reported to act as an anti-angiogenic factor increased in obesity [80], and an anti-angiogenic effect of hypertrophied cells has been reported to operate via TWIST-SLIT2 signaling [81]. Moreover, as further discussed below, hypertrophied adipocytes produce collagen types that can impair angiogenesis [77, 82, 83].

Impaired angiogenesis, originating through effects on the vasculature, on the adipocyte, or on both is likely to be a contributor to metabolic dysfunction associated with adipocyte hypertrophy in obesity [84]. This possibility is supported by findings that experimentally enhancing angiogenesis, for example through enforced overexpression of VEGFA, increases capillary density, reduces adipocyte size, and alleviates the metabolic deficits associated with adipocyte hypertrophy [85–87]. These findings suggest that approaches to improve the angiogenic capacity of hypertrophied adipose tissue in obesity may be of therapeutic value. In support of this view, PPARγ activation has been shown to indirectly elicit angiogenesis [88–90], and nanoparticles that leverage PPARγ activators to elicit angiogenesis in adipose tissue have shown to produce metabolic improvements in mice after experimental overfeeding [91]. Further research to identify pro- and anti-angiogenic mechanisms that accompany adipocyte hypertrophy will help identify additional targets that can elicit similar beneficial effects.

## What do the transcriptomes of adipose tissue cells tell us about angiogenesis?

Multiple cell types are likely to regulate angiogenesis in adipose tissue. While adipocytes occupy most of the volume, they represent less than 50% of the total cell composition of adipose tissue, the remainder including progenitor cells, endothelial cells, fibroblasts and immune cells [92]. The large volume and buoyancy of adipocytes makes them difficult to separate from the stromovascular components, complicating their analysis. Nevertheless, exciting recent progress in single-cell and single-nuclei transcriptomics conducted on adipose depots [93–96], provides a new grasp on potential mechanisms of adipose tissue angiogenesis. It is important to note that these insights are bioinformatically-predicted and are not experimentally proven; nevertheless, they provide a valid framework for deriving new hypotheses on the cellular composition of adipose tissues and on the cell–cell interactions important for function. In this section we explore available single-cell and single nuclei studies with a focus on the data we believe to be relevant to our understanding of adipose tissue angiogenesis and the effects of obesity.

In a recent comprehensive study, Emont et al. provided single cell and single nuclei RNA transcriptomic data from subcutaneous and visceral depots of both humans and mice, allowing inter-depot and inter-species comparisons [93]. Its is important to note that single-cell nuclei RNASeq is restricted to transcripts that remain in the nucleus, thus levels of gene expression in these data sets are influenced by post-transcriptional kinetics and may not reflect total transcript expression for any particular gene. Nevertheless, concordance between single-cell and single nuclei RNASeq is sufficient to allow classification of cells and estimation of cell proportions within the tissue. An interesting aspect of the comparison between mouse and human depots data pertains to the proportion of all cells in adipose tissue that correspond to endothelial cells. The proportion of endothelial cells, as percent of all cells sequenced, appears to be significantly higher in human than mouse adipose tissue (~ 0.2% in human, 0.03–0.06% in mouse tissues). This species-dependent difference may be attributable at least in part to a higher overall infiltration of mouse adipose tissue depots by immune cells. This finding of a comparatively low proportion of endothelial cells (~ 0.01%) in mouse fat was also seen by Sarvari et. al. [97].

Differences in the relative proportion of endothelial cells are also seen between depots within the same species. In humans with BMI < 30, the proportion of endothelial cells relative to all tissue cells was ~ 0.2% in SqAT and ~ 0.07% in visceral adipose tissue, attributable in part by the presence of mesothelial cells in visceral adipose tissue. In both depots, the proportion of endothelial cells relative to all cells in the tissue decreased to approximately half in individuals with BMI > 40 (Emont et al., Extended Data Fig. 1). The observed decrease in the relative proportion of endothelial cells in response to obesity may be relevant to tissue homeostasis, as it may translate into a deficit in perfusion relative to tissue requirements. The lower proportion of endothelial cells is also consistent with capillary rarefaction and microvascular damage in response to obesity reported by other methods [78].

In mice, high fat diet feeding for 13 weeks resulted in large infiltration of visceral (epidydimal) adipose depot by immune cells, with consequent proportional decrease in endothelial cells (Extended Data Fig. 2 in Emont, Jacobs et al.) A similar massive immune cell infiltration after prolonged high-fat diet was seen in study by Sarvari et al. [97]. Sarvari et al. further analyzed mesothelial and endothelial cells, finding that they induced a large proportion of the inflammatory genes induced by high-fat diet [97]. These data highlight that overfeeding has a significant effect on the mouse adipose tissue endothelium, with likely consequences for angiogenesis.

Endothelial cells in human visceral and subcutaneous adipose depots were analyzed in depth by Vijay et al. [96], who analyzed the stromal-vascular fraction of adipose tissue from 12 visceral and 13 abdominal subcutaneous samples derived from 14 individuals with an average BMI of 41 [96]. They identify three types of endothelial cells, one which expressed classical endothelial markers, another expressing genes involved in lipid metabolism, and a third population expressing *LYVE1*, a marker of lymphatic endothelial cells. Their studies reveal that lymphatic vasculature represents 78% of endothelial cells in visceral adipose tissue from obese subjects. Lymphatic vasculature appears in response to inflammation, and its enhancement by genetic overexpression of VEGD in mice decreased inflammation in response to high fat diet feeding [98]. These results suggest that both angiogenesis and lymph-angiogenesis might be affected by obesity, with distinct physiological consequences such as increased inflammation.

Information on the responses of adipocytes to obesity can also be inferred from these studies. Extracted data from Supplementary Table 5 from Emont et al. [93], which represents the average expression of genes in adipocyte clusters from mouse or human depots, either under different dietary conditions (mouse) or from individuals with different BMIs (human) shows 8 adipocyte genes to be decreased in expression level across both visceral and subcutaneous depots of both human and mouse, while no genes are increased across all depots and species (Table 1). The genes that show decreased expression correspond to *ADIPOQ*, *VEGFA*, *IGF1*, *FGFR1*, *MET*, *NAMPT*, *SCTR* and *PRLR*. It is striking that three of these correspond to secreted proteins/growth factors (*ADIPOQ*, *VEGFA*, *IGF1*) which have been directly associated with angiogenesis. Further experiments to confirm that these levels of expression translate into decreased protein secretion from adipocytes are necessary to determine whether these findings might be functionally significant.

Transcriptomic data at single cell resolution can also be used to predict potential interactions between different cell types by examining the expression levels of genes for receptor/ligand pairs. CellPhoneDB is a repository of ligands, receptors and their interactions based on public resources and manual curation of specific families of proteins involved in cell–cell communication, which also considers the subunit architecture for both ligands and receptors to represent heteromeric complexes [99]. Using this resource, Emont et al. [93] report potential interaction networks between cell types in human adipose tissue (Supplementary Table 4 in Emont et al.) including endothelial and progenitor cells. These may be physiologically relevant for angiogenesis given the known perivascular localization of progenitor cells [28–33]. To reveal which predicted molecular interactions might underlie endothelial-progenitor cell networks, we re-analyzed the primary data and find multiple collagen/integrin and growth-factor/receptor pairs classically associated with angiogenesis, but for which very little or no information exists in the context of adipose tissue (Table 2).

Collagens play complex and critical roles in adipose tissue, and collagen composition strongly impacts adipose tissue function [82, 83, 100]. In addition to structural roles, collagens interact with receptors either as full-length proteins or via bioactive fragments released by limited proteolysis [101, 102]. The collagen-receptor pairs predicted to occur on the basis of single cell sequencing offer a window into the role of the extracellular matrix in establishing depot- and metabolic state-dependent adipose tissue functions.

**Fig. 2:**
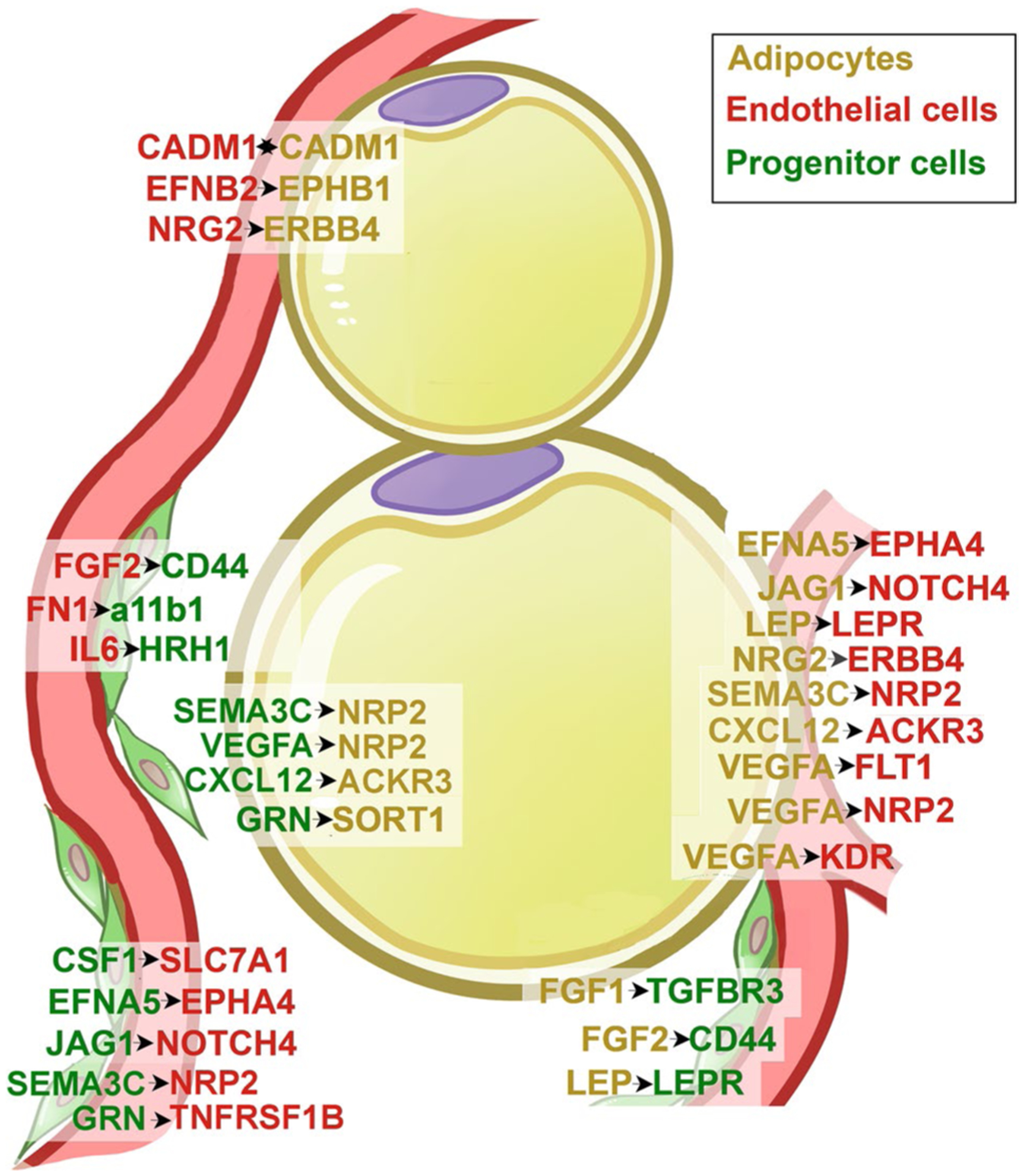
Predicted interactions between adipocytes, endothelial cells and multipotent progenitor cells in human subcutaneous adipose tissue, not including collagens. Cell type interaction analysis was performed using expression data from Emont et al. [93]. The human adipose tissue single-cell/single-nuclei Seurat object (human_all.rds) was retrieved and used to identify the top 5000 most variable features across the dataset using the Seurat FindVariableFeature function. The gene symbol of the top 5000 variable features were translated into Ensembl gene ID using biomaRt (Ensembl Genes 106, hsapiens_gene_ensembl dataset). Normalized count tables for the top features with cells from human SqAT of donors with BMI ≤ 30 or BMI > 40 were exported into tab delimited files for CellPhoneDB analyses. Interaction analysis with CellPhoneDB v3.0.0 was executed in python 3.8.3 environment using the statistical_analysis method and default settings (database release v2.0.0). Gene symbols are depicted in yellow, green or red as a function of their enrichment in adipocytes, multipotent progenitor cells or endothelial cells, respectively. The direction of the interaction (ligand → receptor) is depicted by the arrows

In addition to collagen-integrin networks, predicted interactions between adipocytes, progenitor cells and endothelial cells (Fig. 2) that may be relevant for angiogenesis include interactions between VEGFA in adipocytes and its receptors KDR, FLT1 and NRP2 in endothelial cells, interactions between Jagged1 and Notch4 between adipocytes and endothelial cells, and also between progenitor and endothelial cells. These later could potentially modulate the effects of VEGFA to regulate angiogenesis [103–105]. Another potential signaling pathway that to our knowledge has not been previously associated with adipose tissue involves ephrin (EPH) receptor tyrosine kinases and their ligands (EFN), which mediate cell signaling during normal and oncogenic development [106, 107]. Ephrins and Ephrin receptors can interact with numerous other signaling pathways, for example with the FGFR1 receptor and E-cadherin, potentially affecting angiogenesis. Neuropilin-semaphorin interactions predicted between progenitor cells and adipocytes play critical roles in axonal guidance, and have been implicated in cardiovascular development [108, 109].

Interestingly, the number of predicted cell–cell interactions between endothelial cells, lymphatic endothelial cells, progenitor cells (ASPC), smooth muscle cells, pericytes and adipocytes is higher in adipose tissue from individuals with BMI > 40 compared to < 30 (Fig. 4A in Emont, et al. [93]). We re-analyzed the source data to determine predicted interactions between adipocytes and endothelial cells that underlie these differences (Table 3). The results reveal interactions between collagens and integrins enhanced in obesity, consistent with data obtained previously using other approaches; for example, enhanced levels of COL4A1 have been associated with obesity and correlated with metabolic disease severity [110], and COL6 and its cleavage product endotrophin have been found to affect adipocyte differentiation, lipolysis, and inflammation [83, 100, 101, 111]. Predicted interactions between adipocytes and endothelial cells from obese subjects involve COL4A4, COL4A5 and COL4A3, which are major components of basement membranes [112], and COL11A1, a fibrillar collagen necessary for skeletal development and recently implicated in cancer cell invasiveness [113–115]. A large number of predicted interactions by collagens expressed in endothelial cells appear solely in obesity, including COL1A1, COL1A2, COL3A1, COL5A2, COL6A2, COL6A3, COL12A1 and COL15A1. Interestingly, expression of COL1A1 in endothelial cells is associated with a senescent phenotype [116]. Obesity-induced alterations in collagen type expression in adipocytes and endothelial cells, and the interactions resulting from these changes, may underlie the changes in vascularization seen in obesity [82, 83]. Testing these hypotheses and those involving other signaling pathways in the context of specific cell types is an exciting direction for further study.

## Mechanisms of inter-cellular communication

In addition to well understood endocrine mechanisms involving interaction between secreted hormones and their cell surface receptors, extracellular vesicles have emerged as important mediators of cell-to-cell communication. These structures represent a heterogeneous population of membrane bound vesicles generated via diverse mechanisms. Micro vesicles are generated at the plasma membrane through outward budding and fission, while exosomes are produced inside the cell by inward budding of the endosomal membrane. Exosomes are thus very small vesicular structures contained within multivesicular structures within the endosomal system, which are expelled into the pericellular space upon fusion [117, 118]

The relevance of these pathways in adipose tissue was demonstrated in key studies by Thomous et al. [119], in which mice with an adipocyte-specific knockout of the miRNA-processing enzyme Dicer displayed major decreases in circulating exosomal miRNAs, and revealed adipocytes as a major source of these structures. They also demonstrated functional, inter-tissue communication, where exosomes from brown adipose tissue could modulate expression of genes in liver [119]. Pan et al. [120] reported that secretion of exosomal microRNA-34a by adipocytes inhibits M2 macrophage polarization, resulting in measurable differences in adipose tissue inflammation. Additional support for the physiological relevance of adipose tissue-derived exosomal microRNAs was provided by studies in which the exosome composition in obese mice was found to be different from lean mice, and exosomes from obese mice were reported to induce insulin resistance in lean mice [121]. Moreover, the number and composition of exosomes in human blood is also altered in obesity and in response to bariatric surgery and weight loss [122]. These studies imply that impaired adipose tissue vascularization in obesity may have consequences on systemic exosome transport and metabolic regulation at distal organs. Recent exciting advancements are helping to understand how specific microRNA sequences are involved in cell-specific retention or exosome packaging to mediate their systemic functions [123]

In addition to microRNAs, exosomes and other extracellular vesicles carry lipids, proteins, and other nucleic acids including DNA, mRNA, and long non-coding RNAs [117, 118]. In adipose tissue, exosomal transfer of lipid from adipocytes to macrophages [124] accounted for ~ 1% of adipocyte lipid content per day, and was increased in obese animals. Contents of these exosomes were capable of inducing differentiation of hematopoietic progenitors into macrophage-like cells, revealing their potential to modulate macrophage differentiation and function. In a dramatic example of protein transfer via exosomes in adipose tissue, Crewe et al. found high levels of caveolin 1 protein in adipocytes in which the caveolin 1 gene was knocked out. Despite complete absence of mRNA, the levels of caveolin were high and the protein retained functionality. The presence of caveolin in knockout adipocytes was attributable to exosomal [125] transfer of the protein from endothelial cells. In addition to establishing a mechanisms for endothelial-adipocyte communication, the finding of efficient micro vesicle mediated transfer from endothelial cells to adipocytes suggest that this mechanisms may operate to transfer multiple macromolecular components from the circulation into the adipose tissue interstitial space, through mechanisms involving endocytosis, exosome formation and release. These mechanisms can alleviate the constraint imposed by the tight, non-fenestrated features of the adipose tissue endothelium [126].

Exosome and micro vesicle mediated signaling mechanisms have been specifically implicated in the regulation of adipose tissue angiogenesis. Multiple reports have described pro-angiogenic roles of exosomes derived from adipose tissue progenitor cells [127–131], with some studies identifying exosome-associated microRNAs that could mediate the observed effects. In an early example, Liang et al. [132] found that exosomes secreted by human adipose tissue progenitor cells could be taken up by endothelial cells and promote angiogenesis in vitro and in vivo. Mechanistically, they propose that miR-125a, enriched in exosomes, repressed the expression of angiogenic inhibitor delta-like 4 (DLL4), resulting in enhanced angiogenesis. The finding of miR-125a in exosomes produced by adipose tissue progenitor cells, and its pro-angiogenic effect on human umbilical endothelial cells was confirmed by Pi et al., [133] who also find an effect of these exosomes to inhibit PTEN. In other contexts, miR-125 has been shown to have anti-angiogenic effects [134–136], for example, deletion of miR-125a in zebrafish resulted in a hyper-branching phenotype [134]. Many mechanistic questions related to exosome signaling in angiogenesis remain to be answered, including the regulation of exosome production, exosome composition, and recognition and processing by target cells. The potential for providing a complete understanding of mechanisms of adipose tissue angiogenesis, as well as potential therapeutic uses of exosomes, will continue to fuel this field forward.

## Angiogenesis targeted therapies for obesity: pro- or anti-?

Substantial evidence reviewed above points to the need for angiogenesis for adequate adipose tissue development, needed to prevent lipotoxicity and metabolic disease. Therefore, limiting adipose tissue angiogenesis during the development of obesity would be expected to occur at the expense of metabolic health. Nevertheless, several reports of anti-angiogenic treatment leading not only to lower adiposity, but to improved metabolic parameters exist [137–142] (Table 4). For example, in an early report, improved obesity and metabolism in leptin-deficient ob/ob mice was seen to occur in response to the angiogenesis inhibitor TNP-450 [137]. The drug was introduced into 4–5-week-old mice that had not developed advanced obesity, and resulted in lower body weight gain during the ensuing 12 weeks of treatment. However, the drug also led to a significant decrease in food intake, confounding the interpretation of the role of the TNP-450 anti-angiogenic effect. In complementary studies, While et al. verified the effect of TNP-470 to decrease food intake, and reported an aggravation of glucose tolerance in mice exposed to the drug [143].

Studies on a different anti-angiogenic compound, ALS-L1023 revealed lower weight gain in response to the drug, but this effect was abrogated by pair feeding, revealing decreased food intake as a confounding factor [140]. In a more recent study, Siddik et al. [141] showed that BL6, an inhibitor of methionine aminopeptidase 2 (MetAP2) that impairs endothelial cell proliferation, had additional autonomous effects to inhibit lipid accumulation and stimulate glucose uptake by cultured adipocytes. These results raise the possibility that the effect of small molecule inhibitors of angiogenesis to decrease obesity may be attributable to effects other than those on endothelial cells; further advancement of these therapies will require elucidation of their multiple actions to define those specifically resulting in weight loss [144, 145].

Another class of angiogenesis inhibitors that have been tested in the context of obesity include AARP (CTT peptide-endostatin mimic-kringle 5), a multitarget fusion protein designed against tumor angiogenesis [142]. This drug ameliorated weight gain in C57BL6 mice in response to high fat, high calorie diet, and careful assessments demonstrated that food consumption was unaffected. Strikingly, energy expenditure was significantly higher in mice exposed to the drug, manifested as increased locomotor activity as well as increased temperature. The increase in energy expenditure elicited by AARP could be traced to a strong induction of thermogenic adipocytes in subcutaneous and interscapular depots. Adipose tissue browning is well known to contribute to increased energy expenditure and protection from obesity [146]. Thus, it remains unclear whether the protection from weight gain achieved by AARP treatment is due to its potential anti-angiogenic actions, to its stimulation of adipose tissue browning, or to a combination of both effects.

The pleiotropic effects of inhibitors confound our understanding of the specific consequences of angiogenesis inhibition on obesity and associated metabolic dysfunction. Genetic approaches targeting specific angiogenic mechanisms are less ambiguous, but have also revealed underlying complexity. VEGFA is a potent stimulator of endothelial cell proliferation and angiogenesis, and is expressed by adipocytes and progenitor cells in adipose tissue. Numerous groups have reported that increased expression [85, 86, 147, 148] or enhanced bioavailability [149] of VEGFA in adipose tissue mitigates obesity and its metabolic consequences. In elegant, temporally controlled experiments, Park et al. [85] used a doxycycline-inducible, adipocyte-specific, VEGFA-overexpressing mouse to find that VEGFA overexpression triggered angiogenesis early as 2 days postinduction. However, in all studies angiogenic induction by VEGFA also resulted in formation of thermogenic beige adipocytes, which as mentioned above have cell-autonomous, systemic metabolic effects [36, 85].

Conversely, deficiency in adipose tissue VEGFA signaling results in aggravation of metabolic dysfunction [87, 148]. A role for VEGFB in adipose tissue has also been reported [147]; deletion of adipocyte VEGFB driven by CRISPR-Cas9 resulted in adipocyte hypertrophy, expanded adipose tissue mass, and exacerbation of metabolic dysfunction in response to high fat diet [150]. Conversely, overexpression of VEGFB resulted in amelioration of metabolic effects, and thermogenic induction in adipose tissue, similar to that seen in response to VEGFA [86].

Paradoxically, the use of pharmacological, systemic inhibitors of VEGFA do not recapitulate the metabolic dysfunction elicited by adipose tissue-specific loss of VEGFA; treatment of mice with a neutralizing antibody to VEGFA did not exacerbate the metabolic dysfunction in hyperphagic ob/ob mice, which on the contrary trended to an improvement in glucose levels [151]. Similarly, Wu et al. found that administration of the VEGFA-neutralizing monoclonal antibody B.20–4.1, led to amelioration of high-fat diet-induced insulin resistance, principally due to improved insulin sensitivity in the liver and associated with decreased inflammatory markers [152] These results indicate that systemic inhibition of VEGFA affects multiple organs and tissues leading to improved glucose homeostasis, dominating over the local effect on adipose tissue, which would be expected to impair systemic metabolism.

Genetic models targeting angiogenic factors other than VEGFA and VEGFB support a beneficial effect of adipose tissue angiogenesis independently of browning (Table 4). An et al. [153] found that overexpression of Angiopoietin-2 resulted in enhanced adipose tissue vascularization, decreased fibrosis and inflammation, decreased adipocyte size and improved metabolic parameters in response to high-fat diet, with no detectable induction of thermogenic markers. Conversely, Ang-2 neutralization led to larger adipocyte size and exacerbation of high-fat diet-induced metabolic changes. A similar result was seen in another model, where enhancing endothelial cell proliferation by increasing PI-3 kinase signaling led to enhanced vascularization of adipose tissue and improvements in metabolic parameters [154]. In aggregate, these results suggest that approaches leveraging tissue-specific targeting to elicit angiogenesis in adipose tissue [91] constitute an exciting therapeutic strategy to ameliorate obesity and metabolic disease.

## Conclusions

The increased prevalence of metabolic diseases and their deleterious effects on human health and wellbeing emphasizes the importance of understanding cellular and molecular mechanisms underlying adipose tissue development and its relationship to systemic metabolism. Adipose tissue angiogenesis is at the heart of these mechanisms, as it controls adipocyte metabolism, establishes communication between adipose tissue and the rest of the body, and is critically required for progenitor cell proliferation and tissue remodeling. The application of single-cell and single-nuclei sequencing has opened our horizons on the role of specific signaling pathways operating between endothelial cells, adipocytes and progenitor cells, and their derangements in obesity. Findings revealing new signaling mechanisms consisting of macrovesicles and their contents are also opening up new modes for potential intervention, and results of multiple studies pointing to beneficial effects of enhancing adipose tissue angiogenesis are providing direction for further efforts.

## Figures and Tables

**Table 1:** Genes affected by obesity across depots and species

Gene	Mouse				Human			
	Subcutaneous		Visceral		Subcutaneous		Visceral	
	Normal diet	High fat diet	Normal diet	High fat diet	BMI < 30	BMI > 40	BMI < 30	BMI > 40
ADIPOQ	4.18	1.35	4.66	1.62	9.81	4.93	13.71	5.19
VEGFA	6.11	4.55	6.01	3.42	1.54	1.15	1.43	0.80
IGF1	5.67	4.13	7.44	2.63	4.56	1.21	5.02	0.86
FGFR1	6.60	3.63	5.25	3.23	1.58	1.00	0.99	0.76
MET	1.74	1.02	1.91	1.06	0.18	0.10	0.32	0.14
NAMPT	1.22	0.88	1.09	0.73	1.76	0.54	0.74	0.30
SCTR	2.02	0.31	1.35	0.68	0.42	0.27	0.44	0.29
PRLR	0.35	0.15	3.68	0.93	0.02	0.01	0.04	0.01

Values were extracted from Supplementary Table 5 from Emont et al., representing average expression of genes in adipocyte clusters from mouse or human depots under different dietary conditions (mouse) or from individuals with different BMIs (human) [93]. Genes shown are all decreased by obesity. No genes were found to be increased by obesity across both depots in both species

**Table 2:** Predicted interactions between multipotent progenitor cells and endothelial cells in human subcutaneous and visceral adipose tissues

Progenitor cells to endothelial cells				Endothelial cells to progenitor cells			
SAT		VAT		SAT		VAT	
COL1A2_a1b1	1.079	COL1A2_a1b1	0.568	COL4A2_a11b1	0.434	COL15A1_a11b1	0.181
COL3A1_a1b1	0.881	COL6A3_a1b1	0.537	COL4A1_a11b1	0.415	COL15A1_a1b1	0.127
COL6A3_a1b1	0.685	COL5A2_a1b1	0.458	COL15A1_a11b1	0.277	FGF2_CD44	0.181
COL1A1_a1b1	0.68	COL4A2_a1b1	0.445	COL8A1_a11b1	0.243	FN1_a11b1	0.222
COL6A2_a1b1	0.585	COL6A2_a1b1	0.443	COL21A1_a11b1	0.188	KDR_VEGFC	0.203
COL5A2_a1b1	0.491	COL3A1_a1b1	0.428	CXCL2_DPP4	0.147	TEK_ANGPT1	0.288
COL4A2_a1b1	0.414	COL4A1_a1b1	0.396	FGF2_CD44	0.358	TNFRSF1B_GRN	0.148
COL4A1_a1b1	0.413	COL12A1_a1b1	0.348	FN1_a11b1	0.34		
COL14A1_a1b1	0.347	COL1A1_a1b1	0.32	IL6_HRH1	0.166		
COL12A1_a1b1	0.346	COL15A1_a1b1	0.276	NRP2_SEMA3C	0.348		
COL15A1_a1b1	0.29	COL14A1_a1b1	0.217	TNFRSF1B_GRN	0.196		
COL6A6_a1b1	0.27	COL21A1_a1b1	0.213				
CSF1_SLC7A1	0.303	COL4A5_a1b1	0.213				
EFNA5_EPHA4	0.261	COL6A6_a1b1	0.211				
JAG1_NOTCH4	0.118	COL5A3_a1b1	0.19				
		JAG1_NOTCH4	0.25				
		ACKR3_CXCL12	0.132				

Cell type interaction analysis was performed using expression data from Emont et al. [93]. The human adipose tissue single-cell/single-nuclei Seurat object (human_all.rds) was retrieved and used to identify the top 5000 most variable features across the dataset using the Seurat FindVariableFeature function. The gene symbol of the top 5000 variable features were translated into Ensembl gene ID using biomaRt (Ensembl Genes 106, hsapiens_gene_ensembl dataset). Normalized count tables for the top features were exported into tab delimited files for CellPhoneDB analyses. Interaction analysis with CellPhoneDB v3.0.0 was executed in python 3.8.3 environment using the statistical_analysis method and default settings (database release v2.0.0). Values refer to the total mean of the individual partner average expression values in the interacting pair of cell types. Significant interactions (*P* < 0.05) are distinguished on the basis of the value distribution of all predicted ligand-receptor pairs after random permutation of the cluster labels in all cell types analyzed [99]. a1b1 = Integrin α1β1 complex; a11b1 = Integrin α11β1 complex

**Table 3:** Predicted interactions between adipocytes and endothelial cells in human subcutaneous adipose tissues

Adipocytes to endothelial cells			Endothelial cells to adipocytes		
Interacting_pair	BMI < 30	BMI > 40	Interacting_pair	BMI < 30	BMI > 40
COL4A2_a1b1	0.852	0.888	COL4A2_a1b1	0.499	0.66
COL4A1_a1b1	0.789	0.774	COL4A1_a1b1	0.48	0.625
VEGFA_FLT1	0.587	0.472	TEK_ANGPT1	0.403	0.771
EFNA5_EPHA4	0.571	0.726	NRG2_ERBB4	0.387	0.668
COL5A2_a1b1	0.45	0.671	COL15A1_a1b1	0.342	0.63
COL8A1_a1b1	0.374	0.396	COL8A1_a1b1	0.308	0.571
COL12A1_a1b1	0.3	0.437	EFNB2_EPHB1	0.259	0.309
COL15A1_a1b1	0.276	0.405	COL21A1_a1b1	0.253	0.517
*JAG1_NOTCH4*	0.272		NRP2_SEMA3C	0.25	0.793
COL5A3_a1b1	0.26	0.331	FLT1	0.21	0.243
COL24A1_a1b1	0.256	0.35	NRP2_VEGFA	0.207	0.249
LEP_LEPR	0.226	0.279	CADM1_CADM1	0.121	0.197
VEGFA_KDR	0.21	0.243	**COL3A1_a1b1**		**0.847**
CADM1_CADM1	0.121	0.197	**COL1A2_a1b1**		**0.804**
*NRG2_ERBB4*	0.119		**COL1A1_a1b1**		**0.704**
**FGF1_TGFBR3**		**0.386**	**NRG3_ERBB4**		**0.693**
**FGFR2_EPHA4**		**0.332**	**COL5A2_a1b1**		**0.565**
**COL11A1_a1b1**		**0.304**	**COL6A2_a1b1**		**0.516**
**COL4A4_a1b1**		**0.247**	**COL6A3_a1b1**		**0.504**
**COL4A5_a1b1**		**0.22**	**COL12A1_a1b1**		**0.473**
**COL4A3_a1b1**		**0.218**	**FN1_a11b1**		**0.358**
**VEGFD_KDR**		**0.149**	**GRN_SORT1**		**0.327**
**THBS1_a3b1**		**0.147**	**FLT4_PDGFC**		**0.322**
**VEGFD_FLT4**		**0.079**	**COL15A1_a11b1**		**0.309**
			**EFNB2_EPHA4**		**0.271**
			**COL21A1_a11b1**		**0.196**
			**FGF2_FGFR2**		**0.172**
			**TIMP1_FGFR2**		**0.14**

In bold are interactions that are predicted to occur only in the obese state

Cell type interaction analysis was performed using data from Emont et al. [93]. The human adipose tissue single-cell/single-nuclei Seurat object (human_all.rds) was retrieved and used to identify the top 5000 most variable features across the dataset using the Seurat FindVariableFeature function. The gene symbol of the top 5000 variable features were translated into Ensembl gene ID using biomaRt (Ensembl Genes 106, hsapiens_gene_ensembl dataset). Normalized count tables for the top features were exported into tab delimited files for CellPhoneDB analyses. Interaction analysis with CellPhoneDB v3.0.0 was executed in python 3.8.3 environment using the statistical_analysis method and default settings (database release v2.0.0). Values refer to the total mean of the individual partner average expression values in the interacting pair of cell types. In bold are interactions that are predicted to occur only in the obese state

**Table 4:** Summary of reports on adipose tissue angiogenesis and metabolic outcomes

Authors	Approach	Model	Outcome
Brakenhielm et al. [137]	Pharmacological, TNP-450 (MetAP2 inhibitor)	Ob/Ob mouse	Decreased weight gain, decreased food intake
White et al. [143]	Pharmacological, TNP-450 (MetAP2 inhibitor)	Ob/Ob mouse	Decreased weight gain, decreased food intake, impaired glucose tolerance
Park et al. [140]	Pharmacological, ALS-L1023	Diet-induced obese mouse	Decreased weight gain
Siddik et al. [141]	Pharmacological, BL6 (MetAP2 inhibitor)	Cultured adipocytes	Decreased glucose uptake, decreased lipid uptake
Pottorf et al. [145]	Pharmacological, ZGN-1258 (MetAP2 inhibitor)	Bardett-Beidle mouse model	Decreased weight gain, decreased food intake
Wang et al. [142]	Pharmacological, AARP (CTT peptide-endostatin mimic-kringle 5)	Diet-induced obese mouse	Decreased weigh gain, increased locomotor activity, increased thermogenesis
Robciuc et al. [86]	Genetic, AAV-mediated VEGFB transduction	Diet-induced obese mouse	Improved glucose metabolism, improved adipose tissue vascularization, increased thermogenic adipose tissue
Park et al. [85]	Genetic, increased VEGFA expression through Adiponectin-Cre	Doxicicline inducible, diet-induced obese mouse	Decreased weight gain, increased thermogenic adipose tissue
Jin et al. [147]	Genetic, repressed VEGF expression through AP2-cre	Doxicicline inducible	Decreased weight gain, improved glucose tolerance, improved insulin sensitivity, increased thermogenic adipose tissue
Seki et al. [149]	Genetic, increased VEGFA bioavailabillity in adipose tissue	Anti-VEGFR1 neutralizing antibodies; genetic deletion of VEGFR1	Decreased weight gain, increased thermogenic adipose tissue

## Data Availability

The datasets analyzed for this study were generated by Emont et al. [92], and are publicly available. Methods used to analyze the datasets are described fully within this article.
